# Supplementary material for: Characterization and Genomic Analysis of a Novel Lytic Phage DCp1 against Clostridium perfringens Biofilms
Source: Int J Mol Sci. 2023 Feb 20;24(4):4191. doi: 10.3390/ijms24044191 (PMC9965233; doi:10.3390/ijms24044191)
Supplement: Supplementary file 1 [file ijms-24-04191-s001.zip › Table S1. Lytic activity of phage DCp1 against different strains.pdf]

Table S1. Lytic activity of phage DCp1 against different strains

No.	Strain	EOP	Source
1	C. perfringens BNCC125404	-	BNCC (2020)
2	C. perfringens D1	-	Feces of diarrhea foal in Donge donkey farm, Liaocheng City, Shandong Province, China (2016)
3	C. perfringens D1-2	-	Feces of diarrhea foal in Rongfa donkey farm, Yanggu, Shandong Province, China (2016)
4	C. perfringens D2	-	Feces of diarrhea foal in Donge donkey farm, Liaocheng City, Shandong Province, China (2017)
5	C. perfringens D6	-	Feces of diarrhea foal in Rongfa donkey farm, Yanggu, Shandong Province, China (2017)
6	C. perfringens D9-1	-	Feces of diarrhea foal in Rongfa donkey farm, Yanggu, Shandong Province, China (2018)
7	C. perfringens D10	-	Feces from a donkey with diarrhea in Xinjiang, China (2018)
8	C. perfringens D14	-	Feces of diarrhea foal in Rongfa donkey farm, Yanggu, Shandong Province, China (2019)
9	C. perfringens D22	1.00	Feces of diarrhea foal in Donge donkey farm, Liaocheng City, Shandong Province, China (2019)
10	C. perfringens D23	-	Feces of diarrhea foal in Binzhou donkey farm, Shandong province, China (2019)
11	C. perfringens D30	-	Feces of diarrhea foal in Binzhou donkey farm, Shandong province, China (2022)
12	C. perfringens D31	-	Feces of diarrhea foal in Rongfa donkey farm, Yanggu, Shandong Province, China (2021)
13	C. perfringens CO1	-	Qingdao Nuoanbaite Biotechnology Co., LTD, China (2017)
14	C. perfringens CO2	-	Clinical disease dead chicken, Yantai, China (2017)
15	C. perfringens CO3	-	1-day-old chicks, Yantai, China (2017)
16	C. perfringens CO4	-	Dead embryo, Qingdao, China (2017)
17	C. perfringens CO5	-	Eliminated breeding chicken ovaries, Yantai, China (2017)
18	C. perfringens CO6	-	Clinical disease dead chicken, Weihai, China (2017)
19	C. perfringens CO7	-	Clinical disease dead chicken, Weifang, China (2018)
20	C. perfringens CO8	-	1-day-old chicks, Weifang, China (2018)
21	C. perfringens CO9	-	Eliminated breeding chicken ovaries, Qingdao, China (2018)
22	C. perfringens CO10	0.72	Clinical disease dead chicken, Binzhou, China (2018)
23	C. perfringens C1	-	Eliminated breeding chicken ovaries, Yantai, China (2018)
24	C. perfringens C2	-	Eliminated breeding chicken ovaries, Jinan, China (2018)
25	C. perfringens C3	-	Clinical disease dead chicken, Yantai, China (2018)
26	C. perfringens C10	-	Qingdao Nuoanbaite Biotechnology Co., LTD, China (2018)
27	C. perfringens C28	-	Qingdao Nuoanbaite Biotechnology Co., LTD, China (2019)
28	C. perfringens C30	-	Clinical disease dead chicken, Yantai, China (2019)
29	C. perfringens C38	0.73	1-day-old chicks, Yantai, China (2019)
30	C. perfringens C99	-	Dead embryo, Qingdao, China (2019)
31	C. perfringens C425	-	Eliminated breeding chicken ovaries, Yantai, China (2019)
32	C. perfringens C718	-	Clinical disease dead chicken, Weihai, China (2018)
33	C. perfringens CT1	-	Eliminated breeding chicken ovaries, Qingdao, China (2019)
34	C. perfringens CI1	-	Clinical disease dead chicken, Qingdao, China (2019)
35	C. perfringens CQ21	0.98	Dead embryo, Yantai, China (2019)
36	C. perfringens DO2	-	Qingdao Nuoanbaite Biotechnology Co., LTD, China (2020)

37	C. perfringens DO8	-	Clinical disease dead chicken, Yantai, China (2020)
38	C. perfringens DO20	-	1-day-old chicks, Yantai, China (2020)
39	C. perfringens DO21	-	Dead embryo, Qingdao, China (2020)
40	C. perfringens A1	-	Eliminated breeding chicken ovaries, Yantai, China (2020)
41	C. perfringens P1	-	Clinical disease dead chicken, Weihai, China (2020)
42	C. perfringens DE1	-	Eliminated breeding chicken ovaries, Qingdao, China (2020)
43	C. perfringens F1	0.61	Clinical disease dead chicken, Qingdao, China (2020)
44	C. perfringens M1	-	Dead embryo, Yantai, China (2020)
45	C. perfringens BC1	-	Qingdao Nuoanbaite Biotechnology Co., LTD, China (2021)
46	C. perfringens AL1	-	Clinical disease dead chicken, Yantai, China (2021)
47	C. perfringens WF1	-	1-day-old chicks, Yantai, China (2021)
48	C. perfringens WH1	-	Dead embryo, Qingdao, China (2022)
49	C. perfringens P2	-	Eliminated breeding chicken ovaries, Yantai, China (2022)
50	C. perfringens JC35	-	Dead embryo, Yantai, China (2022)
51	C. perfringens A12	-	Qingdao Nuoanbaite Biotechnology Co., LTD, China (2022)
52	C. perfringens A13	-	1-day-old chicks, Yantai, China (2022)
53	C. perfringens J12	-	Clinical disease dead chicken, Qingdao, China (2022)
54	C. perfringens J16	-	Dead embryo, Qingdao, China (2022)

"-" indicates that phage Lx18 formed no plaque on the agar plate. EOP was determined as the ratio of PFU of DCp1 from each susceptible strain to the pfu of the indicator strain D22.
