# Supplementary material for: Characterization and Genomic Analysis of a Novel Lytic Phage DCp1 against Clostridium perfringens Biofilms
Source: Int J Mol Sci. 2023 Feb 20;24(4):4191. doi: 10.3390/ijms24044191 (PMC9965233; doi:10.3390/ijms24044191)
Supplement: Supplementary file 1 [file ijms-24-04191-s001.zip › Table S2. Antibiotic sensitivity of strains.pdf]

Table S2. Antibiotic sensitivity of strains

No.	Strain	oxacillin	penicillin G	erythrocin	spectinomycin	vancomycin	clindamycin	tetracycline	neomycin	ampicillin	kanamycin
1	C. perfringens BNCC125404	S	R	I	R	S	I	R	I	S	R
2	C. perfringens D1	S	R	I	I	S	S	R	I	I	S
3	C. perfringens D1-2	R	S	S	I	I	R	S	I	I	R
4	C. perfringens D2	S	S	I	I	R	R	I	S	S	S
5	C. perfringens D6	S	I	S	S	R	S	S	I	I	I
6	C. perfringens D9-1	R	S	I	S	S	I	R	S	I	S
7	C. perfringens D10	I	I	S	R	S	S	I	R	S	S
8	C. perfringens D14	R	R	R	S	S	S	I	I	I	S
9	C. perfringens D22	I	I	S	R	R	I	R	I	R	R
10	C. perfringens D23	S	I	I	R	R	S	S	R	I	S
11	C. perfringens D30	S	R	I	I	I	S	S	S	S	S
12	C. perfringens D31	R	S	S	I	I	R	S	I	I	R
13	C. perfringens CO1	S	S	S	R	R	S	S	R	I	I
14	C. perfringens CO2	I	S	S	S	I	I	R	R	I	S
15	C. perfringens CO3	S	R	I	I	S	S	I	I	I	S
16	C. perfringens CO4	S	I	I	R	R	S	R	I	S	S
17	C. perfringens CO5	S	I	I	R	R	R	S	R	S	I
18	C. perfringens CO6	S	I	I	R	R	S	I	S	R	S
19	C. perfringens CO7	I	I	S	R	R	I	R	I	R	R
20	C. perfringens CO8	S	S	I	I	I	R	R	R	I	S
21	C. perfringens CO9	S	I	I	I	R	S	R	R	I	I
22	C. perfringens CO10	S	I	I	R	R	R	R	R	S	S
23	C. perfringens C1	R	S	S	I	I	R	S	I	I	R
24	C. perfringens C2	I	I	I	S	R	R	S	S	I	R
25	C. perfringens C3	I	I	S	R	R	S	I	I	R	S
26	C. perfringens C10	S	I	I	R	R	S	S	S	I	I

27	C. perfringens C28	S	I	I	R	S	R	R	S	I	I
28	C. perfringens C30	S	I	I	S	S	S	R	R	S	I
29	C. perfringens C38	S	R	R	I	I	S	R	I	I	S
30	C. perfringens C99	S	I	I	R	R	R	S	S	I	I
31	C. perfringens C425	R	S	S	I	I	R	S	I	I	R
32	C. perfringens C718	S	R	I	I	S	I	S	I	R	S
33	C. perfringens CT1	I	I	S	R	R	I	R	I	R	R
34	C. perfringens CI1	R	S	S	S	S	I	I	I	I	R
35	C. perfringens CQ21	R	S	S	I	I	R	S	I	I	R
36	C. perfringens DO2	R	S	S	S	I	I	I	S	R	R
37	C. perfringens DO8	I	I	I	S	S	R	R	S	I	I
38	C. perfringens DO20	S	R	R	R	I	I	I	I	S	S
39	C. perfringens DO21	S	S	R	R	I	I	S	I	R	R
40	C. perfringens A1	R	R	I	S	S	I	S	S	S	I
41	C. perfringens P1	S	I	I	S	S	R	R	I	S	I
42	C. perfringens DE1	S	R	R	I	I	R	S	S	I	I
43	C. perfringens F1	S	R	R	I	I	S	S	I	R	S
44	C. perfringens M1	I	I	S	R	R	I	R	I	R	R
45	C. perfringens BC1	R	S	S	I	I	R	S	I	I	R
46	C. perfringens AL1	I	I	I	S	R	R	S	S	I	R
47	C. perfringens WF1	S	R	S	I	S	R	I	S	R	S
48	C. perfringens WH1	S	S	S	S	I	I	R	R	S	S
49	C. perfringens P2	S	R	I	I	S	R	R	I	I	I
50	C. perfringens JC35	S	I	I	R	R	S	S	S	I	I
51	C. perfringens A12	S	I	I	R	S	R	R	S	I	I
52	C. perfringens A13	S	I	I	S	S	S	R	R	S	I

53	C. perfringens J12	S	R	R	I	I	S	R	I	I	S
54	C. perfringens J16	S	R	R	I	I	S	R	I	I	S

"S": sensitivity; "I": medium sensitivity; "R": resistant.
