# Supplementary material for: Characterization and Genomic Analysis of a Novel Lytic Phage DCp1 against Clostridium perfringens Biofilms
Source: Int J Mol Sci. 2023 Feb 20;24(4):4191. doi: 10.3390/ijms24044191 (PMC9965233; doi:10.3390/ijms24044191)
Supplement: Supplementary file 1 [file ijms-24-04191-s001.zip › Table S3. Functional grouping of predicted ORFs in phage DCp1 and their similarity to the database..pdf]

Table S3. Functional grouping of predicted ORFs in phage DCp1 and their similarity to the database.

ORFs	start	stop	strand	Function	Scientific Name	E value	Per.Ident	Accession
ORF1	250	615	366	hypothetical protein CPD7_25 [Clostridium phage CPD7]	Clostridium phage CPD7	3E-76	92.56%	AZF89493.1
ORF2	634	882	249	hypothetical protein vBCPqdyzP5_02 [Clostridium phage vB_CP_qdyz_P5]	Clostridium phage vB_CP_qdyz_P5	1E-40	97.14%	WAX22453.1
ORF3	942	1292	351	hypothetical protein vBCPqdyzP5_03 [Clostridium phage vB_CP_qdyz_P5]	Clostridium phage vB_CP_qdyz_P5	3E-65	98.08%	WAX22454.1
ORF4	1295	1534	240	hypothetical protein vBCPqdyzP5_04 [Clostridium phage vB_CP_qdyz_P5]	Clostridium phage vB_CP_qdyz_P5	2E-30	94.94%	WAX22455.1
ORF5	1536	1862	327	hypothetical protein vBCPqdyzP5_05 [Clostridium phage vB_CP_qdyz_P5]	Clostridium phage vB_CP_qdyz_P5	2E-33	69.16%	WAX22456.1
ORF6	1862	2203	342				None	
ORF7	2215	3846	1632	BppU family phage baseplate upper protein [Clostridium perfringens]	Clostridium perfringens	1E-94	46.53%	WP_243158902.1
ORF8	3885	4913	1029	morphogenesis protein C [Clostridium phage vB_CP_qdyz_P5]	Clostridium phage vB_CP_qdyz_P5	0	99.71%	WAX22459.1
ORF9	5002	5901	900	hypothetical protein vBCPqdyzP5_09 [Clostridium phage vB_CP_qdyz_P5]	Clostridium phage vB_CP_qdyz_P5	0	98.33%	WAX22460.1
ORF10	5913	8309	2397	DNA polymerase [Clostridium phage vB_CP_qdyz_P5]	Clostridium phage vB_CP_qdyz_P5	0	98.87%	WAX22461.1
ORF11	8391	8771	381	hypothetical protein CPD7_15 [Clostridium phage CPD7]	Clostridium phage CPD7	6E-75	96.77%	AZF89483.1
ORF12	9075	9380	306	hypothetical protein vBCPqdyzP5_12 [Clostridium phage vB_CP_qdyz_P5]	Clostridium phage vB_CP_qdyz_P5	1E-58	92.86%	WAX22463.1
ORF13	9400	9678	279	hypothetical protein CPD7_13 [Clostridium phage CPD7]	Clostridium phage CPD7	4E-57	97.83%	AZF89481.1
ORF14	10394	9720	675	endolysin [Clostridium phage vB_CP_qdyz_P5]	Clostridium phage vB_CP_qdyz_P5	2E-163	97.77%	WAX22466.1

ORF15	11261	10527	735	collar protein [Clostridium phage vB_CP_qdyz_P5]	Clostridium phage vB_CP_qdyz_P5	3E-173	99.59%	WAX22467.1
ORF16	12174	11254	921	collar protein [Clostridium phage vB_CP_qdyz_P5]	Clostridium phage vB_CP_qdyz_P5	0	99.67%	WAX22468.1
ORF17	12427	12167	261	hypothetical protein [Clostridium phage vB_CP_qdyz_P5]	Clostridium phage vB_CP_qdyz_P5	2E-53	98.84%	WAX22469.1
ORF18	13735	12452	1284	tail fibers protein [Clostridium phage vB_CP_qdyz_P5]	Clostridium phage vB_CP_qdyz_P5	0	97.89%	WAX22470.1
ORF19	15364	13739	1626	lysozyme-peptidase [Clostridium phage vB_CP_qdyz_P5]	Clostridium phage vB_CP_qdyz_P5	0	99.26%	WAX22471.1
ORF20	16501	15416	1086	hypothetical protein [Clostridium phage vB_CP_qdyz_P5]	Clostridium phage vB_CP_qdyz_P5	0	98.61%	WAX22472.1
ORF21	16779	16513	267	hypothetical protein [Clostridium phage vB_CP_qdyz_P5]	Clostridium phage vB_CP_qdyz_P5	4E-47	93.18%	WAX22473.1
ORF22	17639	16992	648	hypothetical protein [Clostridium phage vB_CP_qdyz_P5]	Clostridium phage vB_CP_qdyz_P5	2E-134	90.99%	WAX22474.1
ORF23	18019	17639	381	hypothetical protein [Clostridium phage vB_CP_qdyz_P5]	Clostridium phage vB_CP_qdyz_P5	9E-75	84.06%	WAX22475.1
ORF24	18186	18031	156	hypothetical protein HWB67_gp25 [Clostridium phage susfortuna]	Clostridium phage susfortuna	2E-21	98.04%	YP_009838564.1
ORF25	18555	18415	141				None	
